# Imprecision Can Promote Illusions of Understanding

**DOI:** 10.1007/s42113-026-00291-x

**Published:** 2026-04-21

**Authors:** Julia M. Haaf, Nicole Cruz, Anne Giacobello

**Affiliations:** Department of Psychology, University of Potsdam, Karl-Liebknecht-Str. 24-25, Potsdam, 14476 Brandenburg Germany

**Keywords:** Understanding, Multilevel modeling, Team science

## Abstract

The essay by Shiffrin, Stigler, and Keil (2026) opens a valuable discussion on the limitations of understanding in scientific practice and theory development. Here, we argue that the essay sometimes lacks precision, which in itself may nurture illusions of understanding. The goal of this commentary is therefore to more precisely discuss three themes from the essay: Regression to the mean, the critique of formal modeling, and the nature of researchers’ limitations. We end with a brief outlook on how illusions of understanding could be mitigated.

## Introduction

In their essay, Shiffrin et al. (2026) argue that scientists are naturally limited in their understanding of observed phenomena. The authors call these limitations illusions and posit that they fundamentally affect scientific practice. The essay is a valuable starting point for a further going discussion about the role of us scientists as human beings in scientific practice. However, at times the essay lacks precision, be it in the definition of terms used or the explanation and scope of core concepts. A prime example is the term *illusion*, which often refers to the misrepresentation of a sensory stimulus. This definition implies the deviation of our perception from the true physical characteristics of a stimulus. It is unclear, however, whether the illusions discussed in the essay are assessed with reference to the true explanation or the true level of uncertainty. Therefore, the term illusion seems elusive.

In some cases, it may be useful to refrain from an attempt to provide an answer to questions that cannot yet be settled on the basis of the limited information available. However, the lack of precision noted here is not due to limited information but a lack of clarity and specificity. This imprecision allows researchers to interpret the essay’s arguments in different ways, potentially leading to divergent conclusions. Much as a vague description of a theory may result in researchers deriving contradictory hypotheses may the essay’s imprecision in itself nurture illusions of understanding. The goal of this commentary is therefore to more precisely discuss three themes from the essay: Regression to the mean, the critique of formal modeling, and the nature of researchers’ limitations. We end with a brief outlook on how illusions of understanding could be mitigated.

## Discussing Regression to the Mean

We felt that the description fell short of explaining the statistical principles in play. Understanding regression to the mean starts with understanding the linear relationship between two variables *X* and *Y*, here illustrated with the height example introduced by Galton and discussed in the essay (see Bland & Altman, 1994a, fordetails). Suppose the average parent height and child height are both 68.2 inches, their standard deviations are 2 and 2.8 inches, respectively, and the correlation is 0.5. Using linear regression to predict child height from parent height, we observe regression to the mean: The slope of the regression line can be calculated as $r\frac{SD_Y}{SD_X} = 0.5\frac{2.8}{2} = 0.7$. If we wanted to predict child height from parent height of 70.2 inches, we would receive $68.2 - 0.7 \times 68.2 + 70.2 \times 0.7 = 69.6$. The value is fewer standard deviations away from the mean of child height than is the value for parent height from its mean. And unless the correlation is perfect, this will always be the case.

There are many contexts in which regression to the mean may occur, and some of them are much more complex than the above example. In those complex, multidimensional settings, the effects become more difficult to characterize, but the underlying idea to avoid misinterpretation remains the same. For psychology (Bland, 1994b) highlight clinical studies were experimental groups are separated based on one measurement (e.g., clinical depression versus baseline based on a depression test) and then compared after treatment on another measurement after treatment; or educational studies where an educational outcome is compared to initial performance (see also Nesselroade et al., 1980). In these cases, regression to the mean makes it impossible to immediately interpret the outcome. While interpretation errors are not uncommon in the literature as Shiffrin et al. (2026) note, proposed fixes are also common. For example, individual change may be more appropriately estimated using a multilevel model (Jones & Spiegelhalter, 2009).

## Criticizing Formal Modeling

Shiffrin et al. (2026) offer two major criticisms of formal models as instantiations of theory. First, researchers may not understand their models fully, and consequently may not be able to comprehensively test the models’ predictions. Second, once model fit is sufficient, researchers may not search for better alternatives.

When it comes to implementing a formal model using software, we may consider different levels of understanding to be sufficient for different purposes. Understanding increases from (1) operational use were software serves as a black box, (2) procedural understanding on following a reasonable workflow, (3) conceptual understanding of the statistical assumptions and inferences, (4) algorithmic understanding of the implemented code, to (5) constructive mastery that allows one to implement all statistical code from scratch. We believe that it is often not necessary to achieve constructive mastery to estimate a formal model. For example, researchers may not understand the implementation of the Hamiltonian Monte Carlo algorithm in the probabilistic programming language Stan (Carpenter et al., 2017), but they may well be able to implement their model in Stan correctly, and evaluate its predictions and the performance of the estimation algorithm (Schad et al., 2021; Veenman et al., 2024). What is crucial, however, is that researchers understand the assumptions, limitations, and appropriate use of the tools they are using. To make progress in science, we have to build on other’s ideas. And software is no exception. This requires an equal amount of trust in other’s abilities and sufficient knowledge to assess whether the software solution performs as intended.

Likewise, we disagree with the notion that modelers may be satisfied with good-enough model fit. Decade-long scientific debates have arisen from small but meaningful model misfit (see, for example, the debate around the law of practice Newell & Rosenbloom, 1981; Heathcote et al., 2000; Evans et al., 2018). One may even argue that modelers care a bit too much about model misfit (Donzallaz et al., 2026; Dutilh et al., 2019). This is exactly what formal models are built to do: To prevent this illusion of understanding by more precisely assessing misfit between a theoretical explanation and data.

Of course, modelers are also prone to cognitive biases and mistakes. To ensure that formal modeling is a reliable scientific tool, robust modeling workflows and principles should be implemented more broadly Lee et al. 2019; Crüwell et al. 2019 and researchers should rely on more than model fit when assessing and comparing the quality of formal models (Roberts & Pashler, 2000; Villarreal et al., 2023).

## Extending Researchers’ Limitations

The illusions of understanding discussed by Shiffrin et al. (2026) focus on cognitive limitations by researchers, which put limits on our understanding and creativity. We think the focus on cognitive limitations is unnecessarily narrow. Researchers are just as much limited by their academic and societal environments. For example, scientists routinely neglect the influence of their prior beliefs and experiences when developing theories and hypotheses, and we can only put concepts and ideas to empirical test that we come up with in the first place (Gould, 2012). What scientists conceive of and decide to study further also depends on the linguistic, cultural and socioeconomic background of both researchers and participants - which in countries with substantive research funding often tends to be somewhat homogeneous (e.g., WEIRD–Western, Educated, Industrialized, Rich, and Democratic, Henrich et al., 2010). Neglecting the impact of one’s own societal context may lead to overgeneralization of research findings, in itself an illusion of understanding. For example, in the study by Segall et al. (1966) *visual* illusions like Muller-Lyer—which is typically considered universal—produced a sizeable effect in a US sample but the effect was nonexistent in a sample of San foragers (Henrich et al., 2010).

Even before the open science movement honed in on the influence of our academic environment, critical scientists have long discussed the influence of society (e.g., presuming that our educated middle class environment is a blank slate with no influence on behavior; see Day et al., 2017; Sabik & Versey, 2024). We believe it is valuable to examine researchers’ limitations and their interactions more holistically when moving forward with this debate.

## Substantiating the Outlook

Speaking of moving forward, we felt the outlook by Shiffrin et al. (2026) only briefly touched on how we should proceed from here. Being "skeptical and humble" (p. 36) is always good advice, but given the extent of issues discussed in the essay perhaps a bit flat. We would like to point to two additional suggestions.

The first suggestion is to build more diverse, interdisciplinary research teams which may lead to fewer blind spots when developing scientific theory and explaining empirical results. While (big) team science is often discussed as a means to share resources (Forscher et al., 2023), it may solve some of the pervasive limitations of understanding (Hoogeveen et al., 2023; Tierney et al., 2020). If research teams discuss honestly and openly, explanations may be more comprehensive and uncertainties may become apparent as different opinions in the team. Of course, collaboration, especially between researchers with different perspectives, can pose challenges. First, to ensure that understanding does not get lost in translation, precision in defining and communicating scientific concepts is absolute key. Second, teams need to be careful that understanding is not outsourced to few team members who hold responsibility while others tag along.

The second suggestion is to embrace uncertainty throughout all components of scientific practice, which may reduce overconfidence in understanding. When embracing uncertainty, we need to critically reflect when we make implicit and explicit decisions in the research process, and report these decisions transparently (Wagenmakers et al., 2021). Uncertainty is not problematic in the advancement of science as long as we approach it with honesty. In any case, working out what we don’t yet understand is already a major step towards increasing understanding and reducing illusions.

## Data Availability

Not applicable

## Data Availability

No datasets were generated or analysed during the current study.
